# Editorial: Deciphering and targeting the mTOR pathway in hematologic malignancies

**DOI:** 10.3389/fonc.2023.1293795

**Published:** 2023-12-01

**Authors:** Luca Lo Nigro

**Affiliations:** Head of Cytogenetic-Cytofluorimetric-Molecular Biology lab, Center of Pediatric Hematology Oncology, Azienda Policlinico “G. Rodolico” – San Marco, Catania, Italy

**Keywords:** mTOR - mammalian target of rapamycin, acute leukemias, PTEN (phosphatase and tensin homolog deleted on chromosome 10), Rapalogs, PI3 K/AKT

The PI3K/AKT/mTOR pathway is an intracellular signaling pathway that plays a pivotal role in translating the detection of extracellular signals into action in a variety of cellular functions. It is directly related to cell cycle progression, proliferation, differentiation, metabolism, autophagy, cancer, and senescence (1). A key upstream regulator of this pathway is the phosphoinositide 3-kinase (PI3K) that catalyzes the conversion of phosphatidylinositol-3,4-biphosphate (PtdIns-4,5- P2 or PIP2) to phosphatidylinositol-3,4,5-trisphosphate (PtdIns-3,4,5-P3 or PIP3), (see **Figure 1**) (2, Feng et al.). A large number of stimuli can active the PI3K, throughout the membrane, phosphorylating PIP2 in PIP3. These lipids serve to recruit proteins such as the serine/threonine protein kinase AKT and the phosphoinositide–dependent kinase–1 PDK1 to the plasma membrane. After its recruitment, AKT is phosphorylated on residues Thr308 and Ser473 and consequently activated by PDK1 and PDK2, respectively (2, Feng et al.).

**Figure 1:**
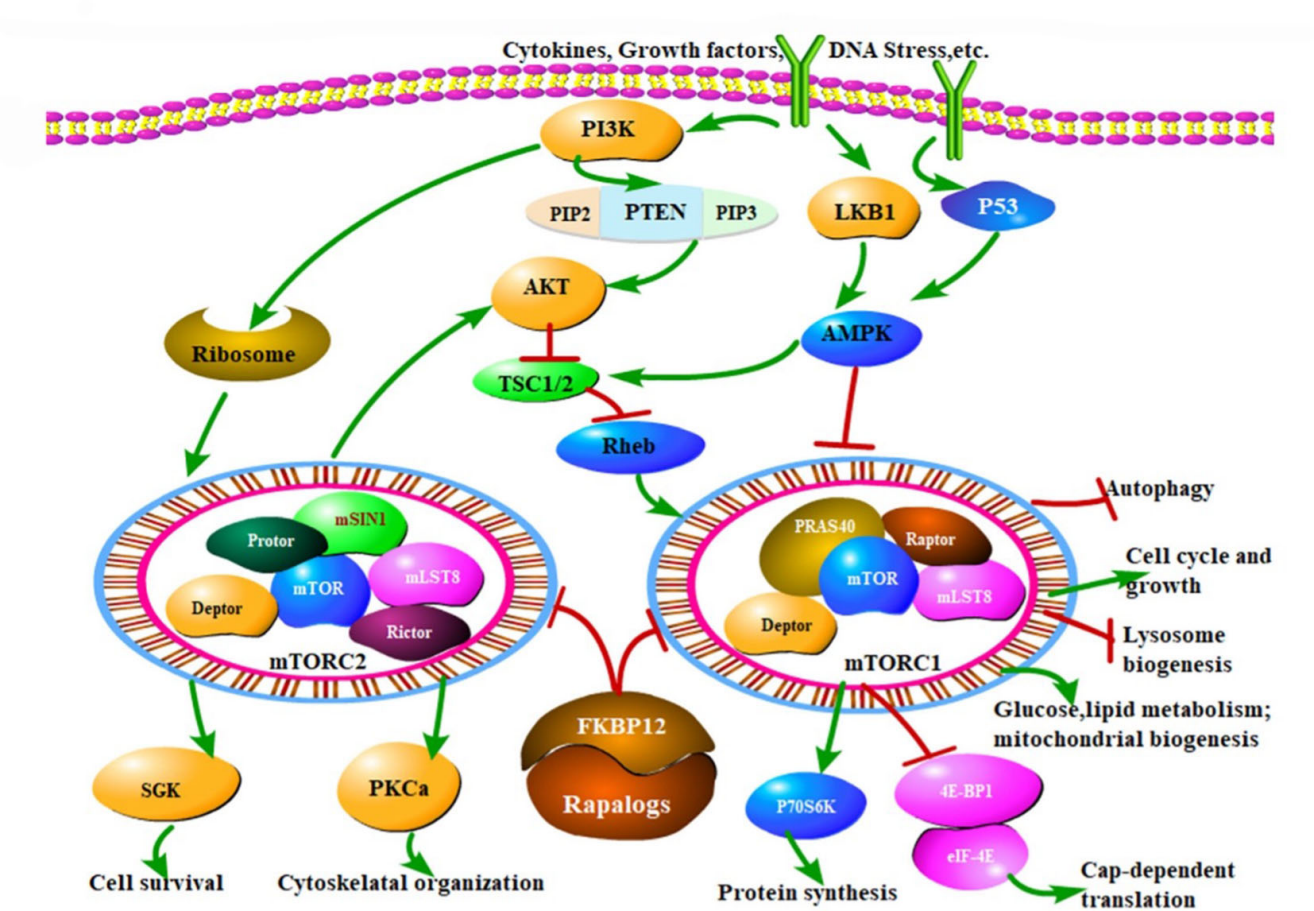
The mTOR signaling pathway and therapeutic mechanisms of drug inhibitors (Rapalogs). MTOR modulates a variety of cellular activities through mTORC1 and mTORC2, responding to extracellular stimuli, such as cytokines, growth factors and DNA stress, which mainly regulate cell growth, cell cycle, and other physiological activities through PI3K/Akt/mTOR pathway. mTORC1 activity is critically inhibited by TSC1/TSC2 complex. PI3K promotes the mTOR activates. Another important pathway of mTOR is regulated by AMPK. Rapalogs can inhibit the kinase activity of mTORC1 and mTORC2, ultimately regulate various biological functions of cells, that is the mechanism of mTOR inhibitors. *Arrows indicate activation and bars represent inhibition*. (Feng Y et al. – Reference 2).

Frontiers in Oncology recently promoted a Research Topic entitled: “*Deciphering and Targeting the mTOR pathway in hematological malignancies*”. Three interesting articles have been published, pointing out different aspects of this pathway. Feng et al., presented a comprehensive review on the regulation and outcome of the mTOR signaling pathways from a basic research perspective, and summarized the use of mTOR inhibitors to target aberrant mTOR activation and signaling in hematological diseases and in the field of blood disorders. They highlighted that perturbation of the mTOR pathway has been shown in several malignant and non–malignant hemopathies: in the former group, including acute and chronic leukemias, lymphomas (Hodgkin and Non–Hodgkin) and multiple myeloma, the mTOR pathway was extensively analyzed and targeted by several mTOR inhibitors, defined as Rapalogs. The most common Rapalogs are: Everolimus (Eve, RAD–001), Deforolimus (Def, Ridaforolimus), Zotarolimus (Zot), and Temsirolimus (Tem, CCI–779).

Among malignant disorders, acute myeloid leukemia (AML) is one of the most heterogeneous diseases. However, the mTOR pathway seems to be extensively implicated (Feng et al., Wu et al). Combination of Rapalogs with other chemotherapies might be an effective regimen for patients with high–risk AML, however, stratification of patients who are likely to respond to these regimens, perhaps based on underlying mutation patterns, must be better understood (Feng et al.). The attention on this disease pointed out on another article presented in the Research Topic. Precisely, Wu et al. demonstrated that deoxyshikonin exerted anti–tumor and anti–glycolytic activities in AML cells by suppressing PKM2 via inactivation of the Akt/mTOR signaling, adding a new promising anticancer candidate agent. The authors started from the basic idea that natural products have the potential to induce apoptosis in cancer cells including AML and may therefore be essential sources for anticancer drugs because of their extensive biological activities and limited side effects. They demonstrated that deoxyshikonin dampened the viability of AML cells in a dose–dependent manner (Feng et al.).

Nevertheless, acute lymphoblastic leukemia (ALL) is an aggressive malignancy of lymphoid progenitor cells in both pediatric and adult patients. Modern genomic approaches have identified several recurrent mutations that can be grouped into several different signaling pathways, including Notch, Jak/Stat, MAPK and PI3K/Akt/mTOR (2). Phosphatase and tensin homolog (PTEN), which acts as a tumor suppressor gene, represents one of the main negative regulators of PI3K/Akt/mTOR network (2), as shown in **Figure 1** (Feng et al.). Inactivating PTEN mutations and activating PI3KCA and AKT1 mutations have been described in numerous malignancies. AKT is a central key regulator of the pathway, and it is involved in a variety of cell function such as glucose metabolism, cell proliferation, and survival. AKT has multiple downstream effectors such as PTEN, GSK3, Caspase–9, BAD, and mTOR2 (**Figure 1**) (Feng et al.). Alterations of PI3K/AKT/mTOR are predominant in T–ALL. Frequent mutational events in T–ALL are detectable in up to 85% Functional analysis of NOTCH1 implicated PTEN in the activation of PI3K/AKT/mTOR mediating resistance to γ–secretase inhibitors (3). Sixteen percent of T–ALL cases harbor mutations or deletions in PTEN leading to PTEN protein deletion (3). Importantly, PTEN is critically involved in maintaining hematopoietic stem cells (HSCs) and preventing leukemogenesis. Several human T–ALL cell lines lack PTEN as a result of deletions or mutations in the gene, with consequent constitutive hyperactivation of the PI3K/Akt/mTOR pathway (3).

In the B–lineage ALL, the Philadelphia Chromosome (Ph) positive ALL is the most common molecular subtype among adults, showing a worst outcome, mainly related to a lack of sustained molecular remission. The mTOR pathway is implicated in the maintenance of the Ph–positive B–lineage ALL cells and the use of new generation of Rapalogs could be of crucial help for reaching the goal of deeper and sustained molecular remission. In the current Research Topic, Lee et al. presented evidence that a third–generation, bi–steric mTORC1 inhibitor (RMC–4627) provides improved anti–leukemia activity compared to rapamycin and a Rapalog compound in models of Ph+ B–ALL. RMC–4627 potently inhibits 4E–BP1 phosphorylation in B–ALL cells *in vitro* at concentrations approximately 8–fold lower than an investigational Rapalog (MLN0128).

In adults, the second most common hematological malignancy is Multiple myeloma (MM), which arises from the uncontrolled proliferation of malignant plasma cells. Despite several effective drugs, currently almost all patients will suffer from a disease recurrence. In this Research Topic, Wang et al. explore the expression of inositol polyphosphate 4–phosphatase II (INPP4B) in MM clinical specimen and cell lines and study the clinical significance of INPP4B and its role in regulating PI3K/Akt/mTOR signaling pathway (7). They elucidated the important role of INPP4B in inhibiting MM cell line proliferation and chemosensitivity *in vitro* and revealed that INPP4B was a low risk factor for MM patient in the clinic. Mechanistically, INPP4B exerted its role through inhibiting PI3K/Akt/mTORc2 signaling pathway.

The current Research Topic focused on the advances in the comprehension of the biological activities and of the impact that mTOR performs in cancer. Significant contribution to the advancement of new therapeutic strategies, aimed to inhibit mTOR, will ameliorate cancer patient outcomes.

## Author contributions

LLN: Conceptualization, Writing – original draft, Writing – review & editing.
